# MRI Findings After Post-abortion Hemorrhage: What if It Were a Uterine Arteriovenous Malformation?

**DOI:** 10.7759/cureus.109665

**Published:** 2026-05-26

**Authors:** El Mahdi Choukri, Youssef Kacimi, Fatima Ezzahra Outoub, Hafsa Lamri, Siham Alaoui Rachidi

**Affiliations:** 1 Diagnostic and Interventional Radiology, Mohammed VI University Hospital, Tangier, MAR; 2 Radiology, Faculty of Medicine and Pharmacy, Abdelmalek Essaâdi University, Tangier, MAR

**Keywords:** enhanced myometrial vascularity, metrorrhagia, pelvic mri, post-abortion hemorrhage, retained products of conception, uterine arteriovenous malformation, uterine artery embolization

## Abstract

Uterine arteriovenous malformations (AVMs) and markedly enhanced myometrial vascularity are uncommon but potentially life-threatening causes of abnormal uterine bleeding, particularly after pregnancy-related events or uterine instrumentation. Their recognition is clinically important because repeat blind uterine evacuation may worsen hemorrhage when a high-flow vascular lesion is present. We report the case of a 35-year-old woman, gravida 5 para 5, who presented to the emergency department 10 days after abortion and uterine curettage with recurrent severe metrorrhagia, hemorrhagic shock, and severe anemia with a hemoglobin level of 7 g/dL requiring transfusion. A pelvic MRI was performed for further evaluation. MRI demonstrated heterogeneous fundal intrauterine material compatible with retained products of conception in the appropriate post-abortion context. This material was closely related to an adjacent abnormal fundal intramyometrial vascular area. Dynamic gadolinium-enhanced sequences showed early enhancement of prominent serpiginous vascular structures within the fundal myometrium and adjacent periuterine vascular channels, raising concern for an acquired uterine AVM-like lesion or markedly enhanced myometrial vascularity associated with retained products of conception. Uterine artery embolization was proposed as a fertility-preserving therapeutic option; however, angiographic confirmation, whether this procedure was performed, and post-treatment follow-up were not documented in the available medical record. This case highlights the value of MRI in severe post-abortion bleeding by clarifying the relationship between retained intrauterine tissue and abnormal myometrial vascularity, while emphasizing the need for cautious terminology when angiographic confirmation is unavailable.

## Introduction

Uterine arteriovenous malformations (AVMs) are uncommon vascular abnormalities characterized by abnormal communications between arteries and veins within the myometrium, without an intervening capillary network. They may be congenital or acquired, with acquired lesions most often described after pregnancy-related events, uterine instrumentation, curettage, cesarean delivery, or trophoblastic disease [1-3]. Although rare, they are clinically important because they may present with abnormal uterine bleeding ranging from intermittent metrorrhagia to life-threatening hemorrhage.

In the post-abortion or post-curettage setting, diagnosis may be challenging because retained products of conception can be hypervascular and may be associated with markedly enhanced myometrial vascularity. This appearance may mimic or coexist with an acquired uterine AVM-like lesion [2-5]. The distinction is clinically relevant because repeat blind curettage in the presence of abnormal myometrial vascularity may increase the risk of major hemorrhage [4,5].

MRI can provide valuable anatomic characterization when the clinical scenario is complex or when a vascular lesion is suspected. It may help define the relationship between intracavitary retained tissue, the myometrium, and adjacent periuterine vascular structures. We report a rare case of severe recurrent post-abortion metrorrhagia in which pelvic MRI demonstrated retained products of conception closely associated with a fundal intramyometrial vascular lesion suspicious for an acquired uterine AVM-like lesion or markedly enhanced myometrial vascularity.

## Case presentation

A 35-year-old woman, gravida 5 para 5, presented to the emergency department on post-abortion day 10 after uterine curettage with recurrent severe metrorrhagia and hemorrhagic shock. Laboratory evaluation showed severe anemia, with a hemoglobin level of 7 g/dL, requiring blood transfusion.

A pelvic MRI was performed for further evaluation of severe post-abortion bleeding and suspected uterine pathology. The case information was compiled retrospectively from the available clinical record and the MRI examination available for review. No CT examination, ultrasound images or report, Doppler data, beta-human chorionic gonadotropin trend, histopathologic documentation, angiographic report, treatment confirmation, clinical outcome, or follow-up imaging were available for review at the time of manuscript preparation. Therefore, the radiologic interpretation was based on the available MRI findings and the documented clinical context.

The MRI protocol available for review included sagittal T1- and T2-weighted imaging, axial fat-suppressed T2-weighted imaging, diffusion-weighted imaging, and dynamic gadolinium-enhanced fat-suppressed T1-weighted sequences acquired during early arterial, early venous, and delayed phases. T2-weighted images demonstrated heterogeneous intrauterine material centered at the fundal region, compatible with retained products of conception in the appropriate clinical context, with adjacent abnormal fundal myometrial signal (Figure 1).

**Figure 1:**
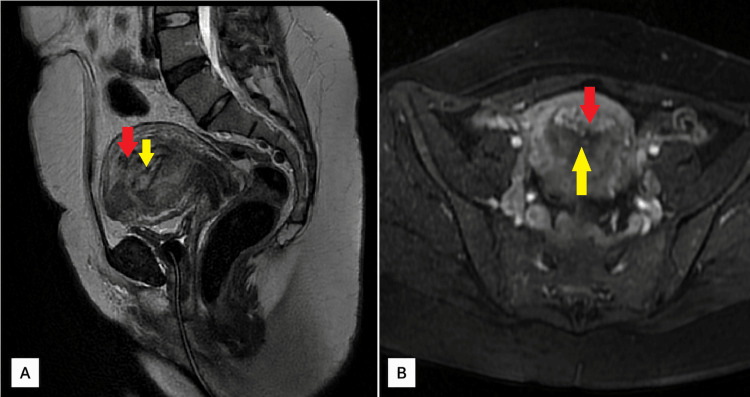
T2-weighted MRI showing fundal retained products of conception and adjacent abnormal myometrial signal. Sagittal T2-weighted image (A) and axial fat-suppressed T2-weighted image (B) demonstrate heterogeneous intrauterine material centered at the uterine fundus, compatible with retained products of conception in the post-abortion clinical context. The retained material is closely related to the adjacent fundal myometrium, where an abnormal signal is seen. The red arrows indicate the intrauterine retained material, and the yellow arrows indicate the adjacent abnormal fundal myometrial region.

Dynamic gadolinium-enhanced images showed early enhancement of prominent serpiginous vascular structures within the fundal myometrium and adjacent periuterine vascular channels, raising concern for an acquired uterine AVM-like lesion or markedly enhanced myometrial vascularity associated with retained products of conception (Figure 2).

**Figure 2:**
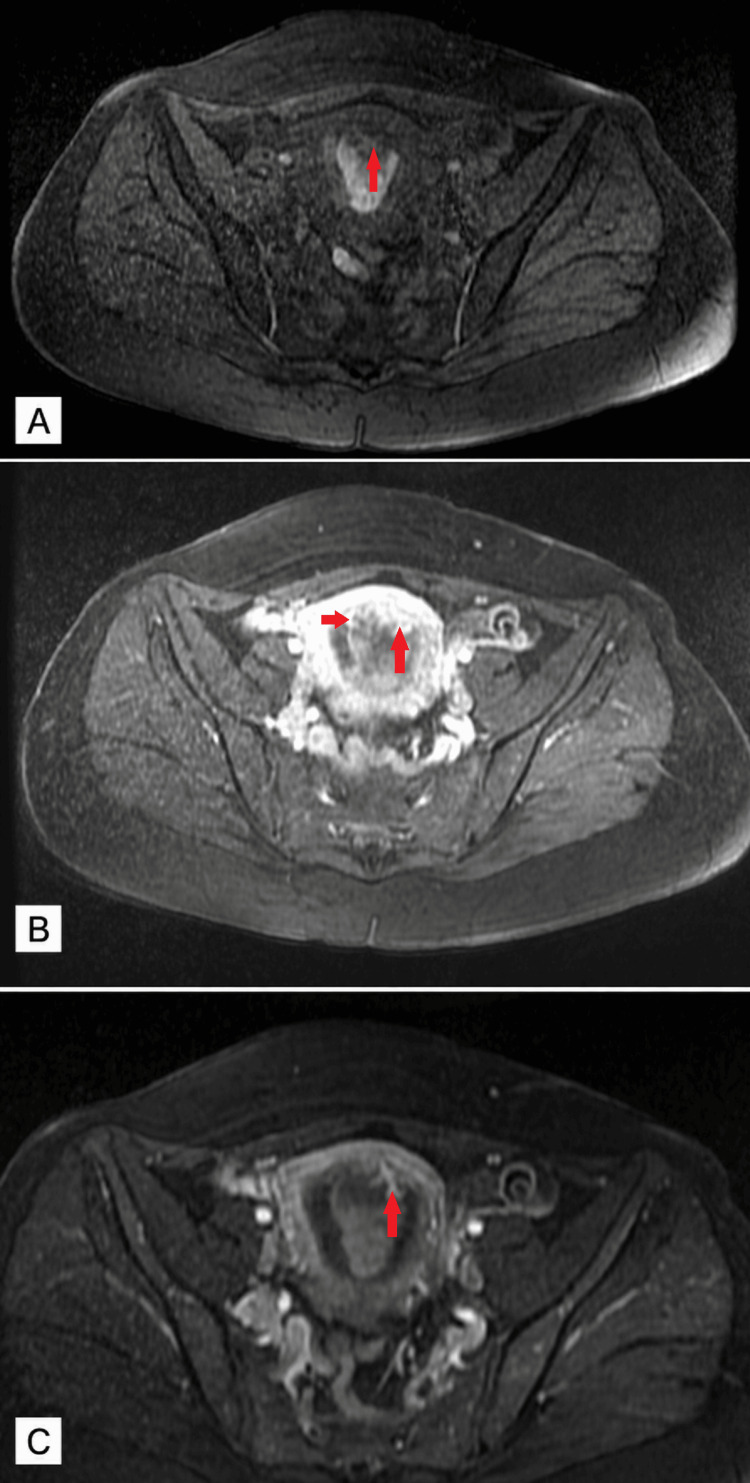
Dynamic contrast-enhanced MRI showing serpiginous vessels of a fundal intramyometrial uterine arteriovenous malformation-like lesion. Early arterial phase (A), early venous phase (B), and delayed phase (C) fat-suppressed T1-weighted images after gadolinium administration demonstrate prominent enhancing serpiginous vessels within the fundal myometrium, adjacent to the retained intrauterine material. The early enhancement pattern raised concern for an acquired uterine arteriovenous malformation-like lesion associated with retained products of conception. The red arrows indicate the enhancing serpiginous vessels of the suspected fundal intramyometrial uterine arteriovenous malformation-like lesion.

Sagittal post-contrast imaging further clarified the close anatomic relationship between the fundal retained intrauterine material and the adjacent abnormal intramyometrial vascular area (Figure 3).

**Figure 3:**
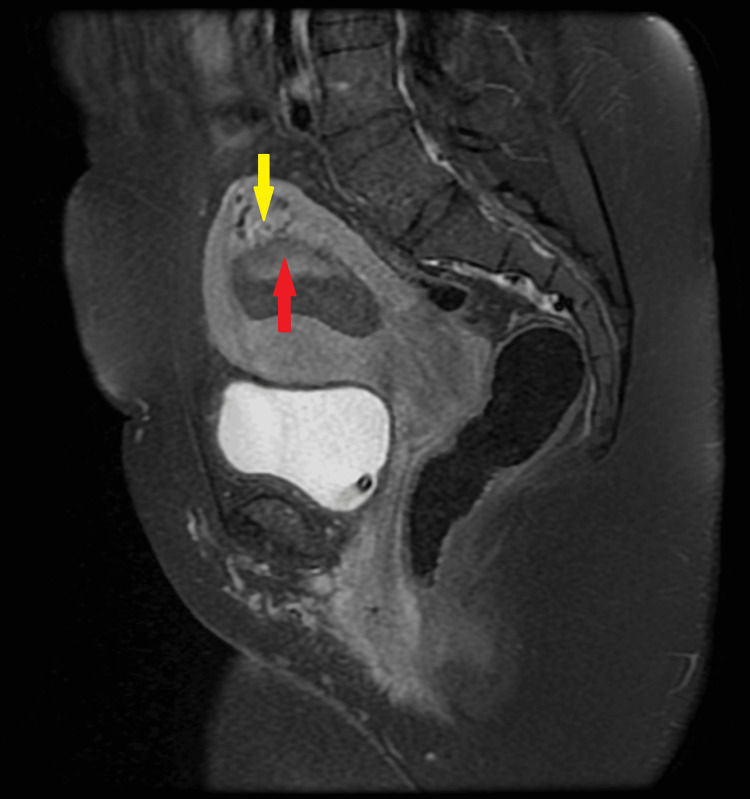
Sagittal post-contrast MRI showing the relationship between retained products of conception and the fundal intramyometrial vascular lesion. Sagittal fat-suppressed T1-weighted image after gadolinium administration demonstrates heterogeneous fundal intrauterine retained material in close contact with an adjacent enhancing intramyometrial vascular area. This anatomic relationship supports the interpretation of retained products of conception associated with an acquired uterine arteriovenous malformation-like lesion or markedly enhanced myometrial vascularity. The red arrow indicates the retained intrauterine material, and the yellow arrow indicates the adjacent enhancing fundal intramyometrial vascular lesion.

Given the severe recurrent bleeding and the MRI suspicion of an acquired uterine AVM-like lesion or markedly enhanced myometrial vascularity, uterine artery embolization was proposed as a fertility-preserving therapeutic option. Angiographic confirmation, whether this procedure was ultimately performed, and post-treatment follow-up were not documented in the available medical record.

## Discussion

The present case illustrates a clinically important diagnostic pitfall in the evaluation of severe post-abortion bleeding. Retained products of conception are a common consideration in this setting; however, when they are associated with abnormal myometrial vascularity or an AVM-like lesion, management may change substantially [5-11]. A vascular lesion should be considered when bleeding is recurrent, severe, or associated with hemodynamic instability, especially after uterine instrumentation such as curettage [1-5,10,11].

The main radiologic issue in this case was the close spatial relationship between fundal intrauterine retained material and an adjacent enhancing intramyometrial vascular area. Hypervascular retained products of conception and enhanced myometrial vascularity can mimic an acquired uterine AVM, and the terminology remains a frequent source of diagnostic ambiguity [5,8-11]. For this reason, the term “uterine arteriovenous malformation” should be used cautiously when angiographic confirmation is unavailable. In the present case, the most scientifically accurate wording is therefore “MRI findings suspicious for an acquired uterine arteriovenous malformation-like lesion or markedly enhanced myometrial vascularity associated with retained products of conception,” rather than a definitively confirmed AVM [10,11].

Although ultrasound with Doppler evaluation is generally the first-line imaging modality for suspected uterine vascular abnormalities and post-abortion hemorrhage, no ultrasound images, Doppler report, or summarized Doppler findings were available for review in this case. This substantially limited the assessment of flow dynamics and diagnostic confidence. MRI was nevertheless useful because it demonstrated both components of the process: heterogeneous fundal intrauterine material compatible with retained products of conception in the clinical context, and adjacent early-enhancing serpiginous vascular structures within the fundal myometrium and periuterine region. This anatomic relationship supported an acquired vascular abnormality or AVM-like enhanced myometrial vascularity rather than an isolated benign uterine mass. The dynamic contrast-enhanced sequences were particularly helpful in demonstrating the early enhancement pattern and the prominence of adjacent vascular channels [3,5,10,11].

The differential diagnosis in this setting included retained products of conception without significant myometrial vascularity, hypervascular retained products of conception, acquired uterine AVM or arteriovenous fistula, uterine artery pseudoaneurysm, subinvolution of the placental implantation site, gestational trophoblastic disease, and leiomyoma with atypical vascularity [4-9,11]. Hypervascular retained products of conception and gestational trophoblastic disease remain particularly important unresolved considerations in this case because beta-human chorionic gonadotropin trends and histopathologic confirmation were not available. In this patient, the post-abortion and post-curettage context, hemorrhagic shock, severe anemia, fundal retained intrauterine material, and adjacent early-enhancing serpiginous myometrial vascular structures favored retained products of conception associated with abnormal myometrial vascularity or an acquired AVM-like lesion. However, in the absence of Doppler, angiographic, histopathologic, and follow-up confirmation, this interpretation should be considered suggestive rather than definitive.

Recognition of abnormal myometrial vascularity is clinically important because repeat blind curettage may expose the patient to severe hemorrhage if a high-flow vascular lesion is present [5,8-11]. In hemodynamically significant cases, uterine artery embolization is commonly considered as a fertility-preserving treatment option when a high-flow uterine vascular lesion is suspected or confirmed [6,7,10,11]. In this case, embolization was proposed because of the severity of bleeding and the MRI suspicion of an acquired vascular lesion; however, the available record did not document angiographic confirmation, the final treatment status, or clinical/imaging follow-up.

This report has several limitations that should be explicitly acknowledged. The case information was compiled from the available clinical record and MRI examination, and several clinically relevant data points were not available for review. As no ultrasound images, Doppler report, or Doppler waveform data were available, ultrasound-based flow characterization could not be included. No CT examination was available. Angiography was not documented, preventing definitive confirmation of arteriovenous shunting. Histopathologic confirmation of retained products of conception, beta-human chorionic gonadotropin trend, confirmation of whether uterine artery embolization was performed, treatment outcome, clinical evolution, and follow-up imaging were also not documented in the available medical record. These limitations reduce diagnostic certainty and justify cautious wording. Therefore, the case is best presented as MRI findings suggestive of an AVM-like lesion or markedly enhanced myometrial vascularity associated with retained products of conception, rather than a proven uterine AVM [10,11].

## Conclusions

Severe post-abortion bleeding should prompt careful evaluation for retained products of conception and possible associated abnormal myometrial vascularity, particularly after uterine curettage and when bleeding is recurrent or associated with hemodynamic instability. This case illustrates how pelvic MRI may help identify retained intrauterine material closely related to abnormal fundal intramyometrial vascularity suspicious for an acquired uterine AVM-like lesion or markedly enhanced myometrial vascularity. MRI can clarify the anatomic relationship between retained intrauterine tissue and abnormal myometrial vascular structures, which may support safer multidisciplinary management. However, because Doppler ultrasound data, angiographic confirmation, treatment confirmation, histopathology, beta-human chorionic gonadotropin trends, and follow-up were unavailable, the diagnosis should remain suggestive on MRI rather than definitive.
